# Diversity in the composition of pleural cavity and oral cavity microbiota in different bacterial empyema

**DOI:** 10.3389/fmicb.2025.1566606

**Published:** 2025-04-29

**Authors:** Yun Huang, Qing-Hua Gao, Chen-Jian Liu, Ting Su, Jie Liu, Zheng-Yi Liang, Zheng-Ju Zhao, Li-Ping Chen, Yong-Ning Yi, Xiao-Ran Li, Jian He

**Affiliations:** Faculty of Life Science and Technology and The Affiliated Anning First People’s Hospital, Kunming University of Science and Technology, Kunming, China

**Keywords:** empyema, pleural cavity, oral cavity, microbiota, similarity

## Abstract

**Introduction:**

Recent studies have proposed primary empyema and demonstrated a correlation between it and the microbial composition of the oral cavity. However, no study has systematically characterized the differences in microbial composition between primary and secondary empyema. Furthermore, the correlation between the characteristics of empyema and oral microbiota remains to be explored.

**Methods:**

The study included forty-six patients diagnosed with empyema. Hydrothorax was collected from all patients, and mouthwash samples were collected from 24 patients. Both types of samples underwent amplification and sequencing using primer sets specific for the 16S rRNA gene.

**Results and discussion:**

Compared with the primary empyema group, the pleural cavity microbial diversity of pneumonia complicated with empyema was significantly decreased (p < 0.05). At the phylum level, the relative abundance of Proteobacteria was significantly higher in the primary empyema group than pneumonia with empyema (p < 0.05). At the genus level, the abundance of *Streptococcus*, *Escherichia-Shigella*, and *Corynebacterium* increased in the primary empyema group, while the abundance of *Campylobacter*, *Salmonella*, *Bacillus*, and *Staphylococcus* decreased (*p* > 0.05). The shared sequences between the hydrothorax samples and mouthwash samples from the patients with empyema contributed to 94% of the total sequences used in these analyses. Correlation analysis indicated that the presence of *Streptococcus constellatus* in empyema is positively correlated with leukocytes and neutrophils, and negatively correlated with lymphocytes (*p* < 0.05).

## 1 Introduction

Empyema was defined as a severe infection characterized by the presence of bacteria or pus in the pleural cavity, associated with high morbidity and mortality (Bedawi et al., 2023). Traditionally, bacterial pneumonia has been considered the primary cause of empyema, where bacteria breach the visceral pleura and form infectious parapneumonic effusion (Corcoran et al., 2015; Shen et al., 2017). However, this concept has been challenged in recent years. Studies have shown that pneumonia and empyema differ microbiologically (Hassan et al., 2019; Kanellakis et al., 2022), and one-third of patients with pleural infections lack the radiological features of pneumonia (Franklin et al., 2021). This subgroup of patients was referred to as “primary empyema” (PE). PE and two types of secondary empyema are the three most common types of empyema in clinical practice. Some other empyema cases were caused by pulmonary abscess, these patients and empyema secondary to pneumonia were collectively referred to as “secondary empyema.” In clinical practice, the complex etiology and variable microbiological characteristics of empyema continue to pose significant challenges for its treatment.

The treatment of empyema critically depends on the timely drainage of hydrothorax and the initiation of targeted antimicrobial therapy. However, pathogen identification through culture-based testing, considered the “gold standard” has a positivity rate of only 56% (Hassan et al., 2019), and only one pathogen can be detected. With the widespread adoption application of novel molecular biology detection technologies such as metagenomic next-generation sequencing (mNGS), the positive detection rate of pathogens in empyema has rapidly increased. This has led to a deeper understanding of the complex pathogen composition in empyema, with most cases involving multiple pathogens.

Notably, anaerobic bacteria are frequently identified and appear to have a close association with the oral microbiota. Through 16S rRNA gene sequencing analysis, Dyrhovden et al. revealed significant microbial community overlap between empyema fluids and oral cavity samples suggesting a possible oral origin that spreads to the pleural cavity via hematogenous dissemination (Dyrhovden et al., 2019). Katsuda et al. (2022) showed that genetically identical strains were present both lung abscess and empyema pus samples, as well as oral swab samples. However, current research on this topic has been limited by small sample sizes and insufficient clinical studies, resulting in a very limited understanding of the microbial composition in pleural infections. To date, only a limited number of studies have concurrently analyzed the microbiota of both the oral cavity and pleural cavity, and there is a notable lack of reports comparing these microbial communities based on the etiology or subtypes of empyema.

The purpose of this study is to utilize 16S rRNA sequencing technology to analyze the differences in microbial composition among various types of community-acquired empyema, including primary empyema, pneumonia combined with empyema, and lung abscess combined with empyema. It also investigates the similarities between the microbial compositions in the pleural cavity and the oral cavity of patients with different types of bacterial empyema. This is the first systematic study to investigate the microbial composition of different types of empyema in adults.

## 2 Materials and methods

### 2.1 Patients and study design

Empyema was defined as Bedawi et al. (2023): pleural collection in the context of infective symptoms with a pH < 7.2, or a low glucose (< 2.2 mmo/L, in the presence of normal serum blood glucose), a pleural collection that is culture positive. The patients were divided into three groups based on the underlying aetiology: primary empyema (PE), pulmonary abscess-related empyema (PARE), and pneumonia with empyema (PWE), in which the latter two groups belonged to “secondary empyema.” The type of empyema was distinguished according to the secondary medical history and clinical manifestations, such as fever, cough, purulent sputum, dense inflammation, and radiological features. PARE (Cai et al., 2019): Chest CT showed lung fields, including visible cavities, fluid levels, and all patients had a lung abscess spreading or rupturing resulting in pus accumulation in the thoracic cavity (WBC ≥ 10 **×** 10^4^/L). PWE (Metlay et al., 2019): Chest imaging showed emerging patchy infiltrating shadows, lobar or segmental solid shadows, ground glass shadows, or interstitial changes with or without pleural effusion. To reduce the impact of the hospital environment, strict negative controls were set up for all samples, which were collected and dispensed in a sterile environment. All patients provided hydrothorax, and 24 of them also provided mouthwash samples. Only the first visit sample was used for all analyses. The missing baseline data rate was 4.8%, which was mainly due to the inability of some patients to provide complete medical history information. Twenty-two mouthwash samples were discarded because patients received antibiotics prior to admission. We utilized a deletion method and exclusively analyzed individuals with no missing data. As this was a between-group comparison, missing data had little impact on the primary outcome.

The study was approved by the Ethics Committee of the Affiliated Anning First People’s Hospital of Kunming University of Science and Technology (2022–025-01). This study complies with the ethical principles outlined in the Helsinki Declaration, and all patient-related data were treated with strict confidentiality.

Exclusion criteria included: (1) Patients aged <18 years; (2) Non-infectious hydrothorax; (3) Hydrothorax caused by tuberculosis or other non-bacterial pathogens; (4) Hospital-acquired empyema.

### 2.2 Sample collection

This study consecutively included 46 hydrothorax samples and 24 mouthwashes in Anning First People’s Hospital Affiliated to Kunming University of Science and Technology from May 2021 to July 2023. Hydrothorax was obtained through thoracentesis drainage or medical thoracoscopy and was subjected to routine biochemical checkout and microbiological culture. Confirm that the patient has not used antibiotics before collecting the sample, avoid eating, drinking or brushing teeth for 1–2 h, use distilled water as a mouthwash under the supervision of a doctor and spit the mouthwash into a sterile collection tube at the end of the rinse. All samples were delivered to the laboratory for processing within 2 h of collection to ensure sterility and timely testing. To detect contamination in the sampling process and the experimental process, strict negative controls were carried out. When each hydrothorax was sampled, two blank centrifuge tube were placed and mock sampled at the same time. For samples placed in centrifuge tubes, approximately the same amount of sterile water was placed in the same batch of sterile single-use collection tubes. All negative control samples were also used in the subsequent experimental process.

### 2.3 Hydrothorax and blood routine measurements

In the study cohorts, biochemical measure ments on hydrothorax samples were conducted with fully automatic biochemical analyzer Hitachi 7,180 (Hitachi High-Technologies, 7,180) using standard photometric methodologies and according to the manufacturer’s instructions. Specifically, High-sensitivity C-reactive protein was measured by latex-enhanced immunoturbidimetry using a high-sensitivity C-reactive protein assay kit (Bio-Technologies Co., Ltd., China). The total protein in was measured by the total protein assay kit (Mindray Biotechnology Co., Ltd., China) using the biuret method. The albumin was measured by the albumin assay kit (Mindray Biotechnology Co., Ltd., China) using the bromocresol green method. The lactate dehydrogenase was measured by the lactate substrate method. The glucose was measured by the glucose assay kit (Mindray Biotechnology Co., Ltd., China) using the hexokinase method. Blood cells were counted by sheath flow impedance cytometry on a Mindray BC-6800 flow line (Mindray Bio-Medical Electronics Co., Ltd., BC-6800) to measure leukocyte, neutrophil, and lymphocyte counts. CD4^+^ T counts were measured by Beckman Coulter CD45/CD4/CD8/CD3 detection kit (Beckman Coulter, Inc., United States) on a DxFLEX flow cytometer (Beckman Coulter Bio-Technology Co., Ltd., DxFLEX). Calcitonin was measured by chemiluminescent immunoassay using a procalcitonin assay kit (Mindray Bio-Medical Co., Ltd., China) on a Mindray CL-2000i fully automatic chemiluminescent immunoassay (Mindray Bio-Medical Electronics Co., Ltd., CL-2000i).

### 2.4 DNA preparation, PCR amplification, and sequencing

Sample processing and DNA extraction work was meticulously performed in a strictly controlled and sterile environment. The experiment was set up with strict negative control, and the laboratory sterile water was selected as the control to participate in the subsequent experiments. All 1.5 mL of the hydrothorax and mouthwash samples were centrifuged for10 min at12000 r/min, and the pellet was used for DNA extraction. Microbial DNA was extracted all of samples using the QIAamp Pro Prowerfecal DNA Kit (Qiagen, Hilden, Germany) according to the manufacturer’s protocol. DNA yield was assessed using Nanodrop 2000 (Thermo Fisher, United States). Primers 515F (Reysenbach et al., 1992) and 909R (Brunk and Eis, 1998) were used for PCR amplification. All primers used containing Illumina adapter sequences and dual-index barcodes to distinguish each sample. For each PCR reaction, 15 ng DNA was added as template. The PCR reaction conditions were as follows: predenaturation at 95°C for 15 s, followed by 30 cycles of 95°C for 3 min, annealing at 51°C for 30 s, extension at 72°C for 30 s, then a final extension step at 72°C for 5 min. During the DNA extraction and PCR batch for each sample, the same reagents and consumables were used, and the PCR amplification procedure was the same for all DNA. No PCR bands were detected in all negative control samples. The amplicon were purified with UltraClean PCR Cleanup Kit (MOBIO, United States), and the equivalent amount of PCR products were sequenced in the Illumina Miseq™ system (Illumina, United States).

### 2.5 Sequence analysis and statistics

All sequences were demultiplexed using the barcodes of each sample. The standardization of the sequences was processed using Mothur v 1.42.0 (Schloss et al., 2009) according to MiSeq SOP. SILVA (V138) (Pruesse et al., 2007) database was downloaded from Mothur website for sequence alignment and classification. Chimerism checking was performed after sequence alignment. Operational taxonomic units (OTUs) were clustered according to the minimum homology threshold of 97%. Measurements of *α*-diversity (within sample diversity) such as observed, Shannon, Simpson ACE, Chao1 indexes and OTU numbers were calculated at OTU level using the Mothur. GraphPad was used for statistical analysis and *p*-value was set to be 0.05. The analysis of *β*-diversity (diversity between samples) was calculated by the Bray Curtis dissimilarity measure with Mothur and expressed by principal component analysis (PCA). Linear Discriminant Analysis (LDA) effect size (Gao et al., 2024) was used to identify the differential OTUs responsible for the discrimination between the differential groups.

Clinical data were analyzed using SPSS 22.0 and GraphPad Prism 9 software. Continuous variables with normal distribution were represented by mean **±** standard deviation (mean **±** SD), and data were compared by independent sample *t*-tests or chi-square test, count data are expressed as percentage (%), *p* < 0.05 was statistically significant. Correlations between the variables were assessed by the Pearson’s correlation coefficient.

## 3 Results

### 3.1 Description of study population

The 46 participants (21 primary empyema, 11 pulmonary abscess-related empyema and 14 pneumonia with empyema) were included in the study Table 1. All patients provided hydrothorax, and 24 of them also provided mouthwash samples. The mean age of the participants was 59 years (SD **±** 13.2), and 87% (40) were male participants. In the case of similar height, the BMI of the primary empyema group was higher than that of the secondary empyema. Patients from the PWE group were significantly younger and possessed higher lactate dehydrogenase levels than those of the other two groups (*p* < 0.05). CD4^+^ was significantly higher in the primary empyema group than in the secondary empyema (*p* < 0.05). Overall, these data suggest that patients with primary empyema exhibit clinical features significantly different from secondary empyema patients.

**Table 1:** Baseline characteristics of participants.

Number	Primary empyema(21)	Secondary empyema (25)		*P*
		PARE(11)	PWE(14)	
Mean age (year)	59.7 ± 13.8	54.9 ± 12.8	60.6 ± 13.7	< 0.05^a^
Male	20 (95%)	10 (91%)	10 (71%)	
Height	1.7 ± 0.08	1.6 ± 0.06	1.6 ± 0.08	
BMI	22.96 ± 3.19	21.84 ± 3.31	22.02 ± 4.38	
C-reactive protein (mg/L)	167.8 ± 99.9	119.9 ± 130.0	135.8 ± 114.4	
Procalcitonin (ng/mL)	0.9 ± 0.70	16.5 ± 37.9	12.5 ± 22.7	
White blood count (10^9^/L)	15.4 ± 8.8	12.8 ± 7.9	14.4 ± 10.6	
Ratio of neutrophils	83.3 ± 6.9	53.9 ± 35.4	71.5 ± 26.3	
Ratio of lymphocytes	9.7 ± 5.3	10.9 ± 6.7	13.0 ± 9.8	
CD4^+^	419.3 ± 289.0	298.7 ± 170.7	214.9 ± 152.1	< 0.001^b^
B lymphocyte	14.2 ± 8.8	19.5 ± 18.6	16.8 ± 8.1	
PH	7.4 ± 0.4	7.1 ± 0.8	7.5 ± 0.08	
Nucleated red blood cel	26879.9 ± 26878.5	93166.6 ± 141756.2	48660.4 ± 60123.0	< 0.05^b^
Lactate dehydrogenase (U/L)	1983 ± 1368.0	3036.77 ± 2695.0	2516.1 ± 2547.8	< 0.05^b^
Total Protein (g/L)	44.4 ± 9.6	36.7 ± 15.8	40.3 ± 16.2	
Glucose (mmol/L)	2.92 ± 2.83	4.00 ± 3.99	2.92 ± 2.88	

Data are presented as *n* (%) or means ± SD. PARE, pulmonary abscess-related empyema, PWE, pneumonia with empyema.

a Statistically significant difference between the primary empyema and pulmonary abscess-related empyema.

b Statistically significant difference between the primary empyema and secondary empyema.

### 3.2 Distinct microbial abundance and composition within primary empyema and secondary empyema

**Figure 1:**
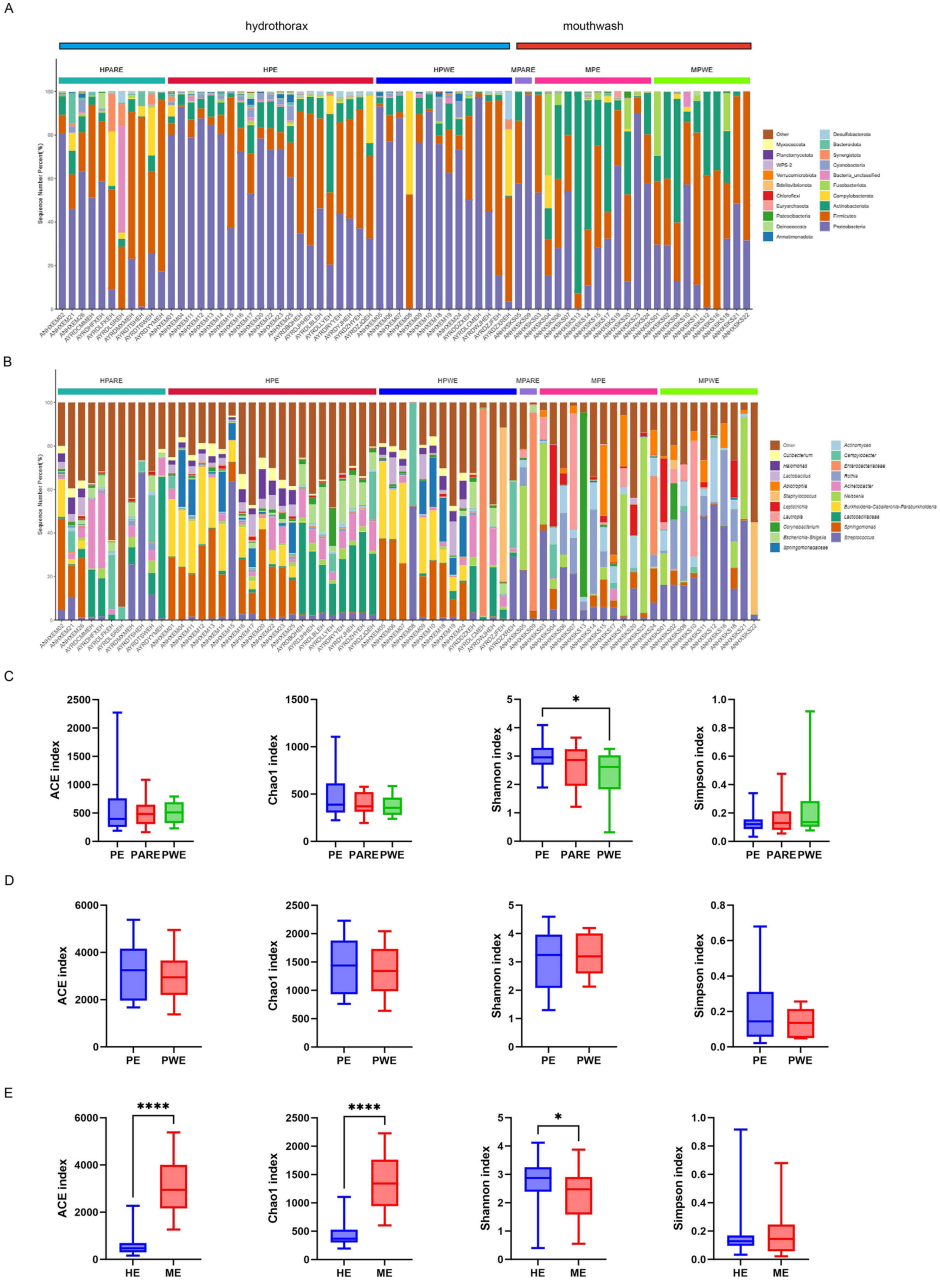
Microbial composition and diversity analysis of the pleural cavity and the oral cavity. **(A)** The composition ratio of each microorganism at phylum and **(B)** genus level. HPARE, hydrothorax–pulmonary abscess-related empyema; HPWE, hydrothorax–pneumonia with empyema; HPE, hydrothorax –primary empyema; MPARE, mouthwash –primary empyema; MPWE, mouthwash–pneumonia with empyema; MPE, mouthwash –primary empyema. **(C)** Alpha diversity between different groups was calculated in hydrothorax based on ACE, Chao1, Shannon and Simpson indices. **(D)** Alpha diversity between different groups was calculated in mouthwash. **(E)** Alpha diversity between hydrothorax and mouthwash were calculated in same groups. *t*- test, *p* values represented as ^*^p < 0.05, ^**^p < 0.01, ^***^p < 0.001.

The relative abundance of different samples at the phylum and genus level was described in Figures 1A,B. The majority of sequences belonged to Proteobacteria (35.5%), Firmicutes (26.1%), and Actinobacteriota (20.6%), which collectively accounted for more than 82% of all the sequences. In hydrothorax samples, the abundance of Proteobacteria was significantly higher in the PE group (56.9 ± 5.3%) compared to the PARE group (34.2 ± 6.8%) (*p* < 0.05). In mouthwash samples, the abundance of Proteobacteria in primary empyema (38.2 ± 7.2%) was much higher than in secondary empyema (26.7 ± 3.6%), while the abundance of Fusobacteria (4.9 ± 1.2%) was similar. At the genus level, the primary empyema group showed an increase in *Escherichia-Shigella* (6.2 ± 8.4%) and *Corynebacterium* (2.2 ± 3.6%), while the genera *Campylobacter* (2.3 ± 6.2%), *Salmonella* (0.3 ± 3.2%), and *Staphylococcus* (0.2 ± 1.3%) decreased in hydrothorax samples. In the PARE group, the genera *Campylobacteria* (5.9 ± 9.6%) were more prevalent, where their abundance was three times that of primary empyema (2.3 ± 6.2%). In PWE group, there genera significantly increased abundances, including *Burkholderia* (10.0 ± 11.3%), *Salmonella* (7.0 ± 25.9%) and *Staphylococcus* (5.5 ± 20.1%), while those of three genera significantly decreased, including *Escherichia-Shigella* (2.3 ± 4.1%), *Bacillus* (1.2 ± 2.5%) and *Neisseria* (0.7 ± 1.6%). In the mouthwash samples, the abundance of the genera *Enterobacter* (7.0 ± 24.2%), *Rothia* (10.0 ± 13.7%) and *Staphylococcus* (3.7 ± 11.4%) in the PWE group higher than the PE group. The abundance of the genera *Neisseriaceae* (6.4 ± 16.6%) and *Corynebacterium* (7.2 ± 23.8%) was higher in the PE group than the PWE group.

Through the comparison of *α*-diversity index (ACE, Chao1, Shannon and Simpson indices), it was found that the microbial diversity index varied in the empyema subgroup. The microbial diversity of hydrothorax was higher in the primary empyema group than in the secondary empyema group, while the Shannon index of PE group was significantly higher than PWE group (*p* < 0.05) (Figure 1C). The diversity index of mouthwash was no statistically significant differences between primary empyema group and secondary empyema group (Figure 1D). The diversity index of hydrothorax was significantly lower than mouthwash samples (*p* < 0.05) (Figure 1E).

### 3.3 OTU-based analysis of different samples in primary and secondary empyema

**Figure 2:**
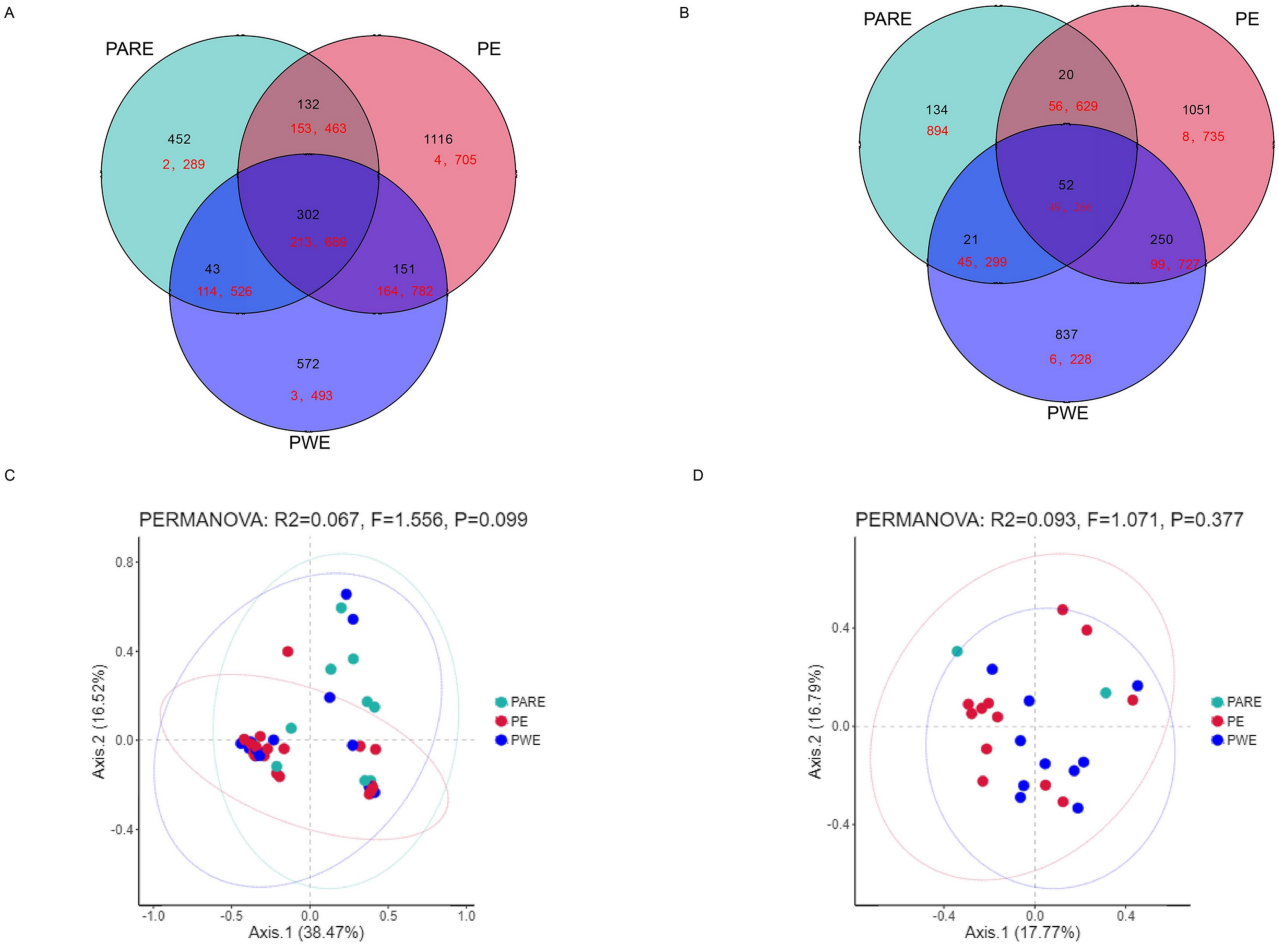
Differences in microbial diversity of pleural cavity and oral cavity among the PE patients, the PWE patients and PARE patients. **(A)** Venn diagram shows the OTUs and sequences shared by different groups at hydrothorax. **(B)** Venn diagram shows the OTUs and sequences shared by different groups at mouthwash. Black shows the OTU number; red shows the sequence number. **(C)** PCA plot based on the relative taxon abundance in hydrothorax samples from different groups. **(D)** PCA plot based on the relative taxon abundance in mouthwash samples from different groups.

Therefore, the difference in the bacterial community in the hydrothorax samples and mouthwash samples between the PE group, PARE group and PWE group deserves further study. First, the shared sequences between the PE group, PARE group and PWE in the hydrothorax contributed 95% of the total sequences used in these analyses (213,689 raw sequences), and the percentage from mouthwash samples was 32% (Figures 2A,B). As shown in Figures 2C,D, a principal component analysis (PCA) found that the primary empyema could not be distinguished from the secondary empyema group by its respiratory tract microbial.

**Figure 3:**
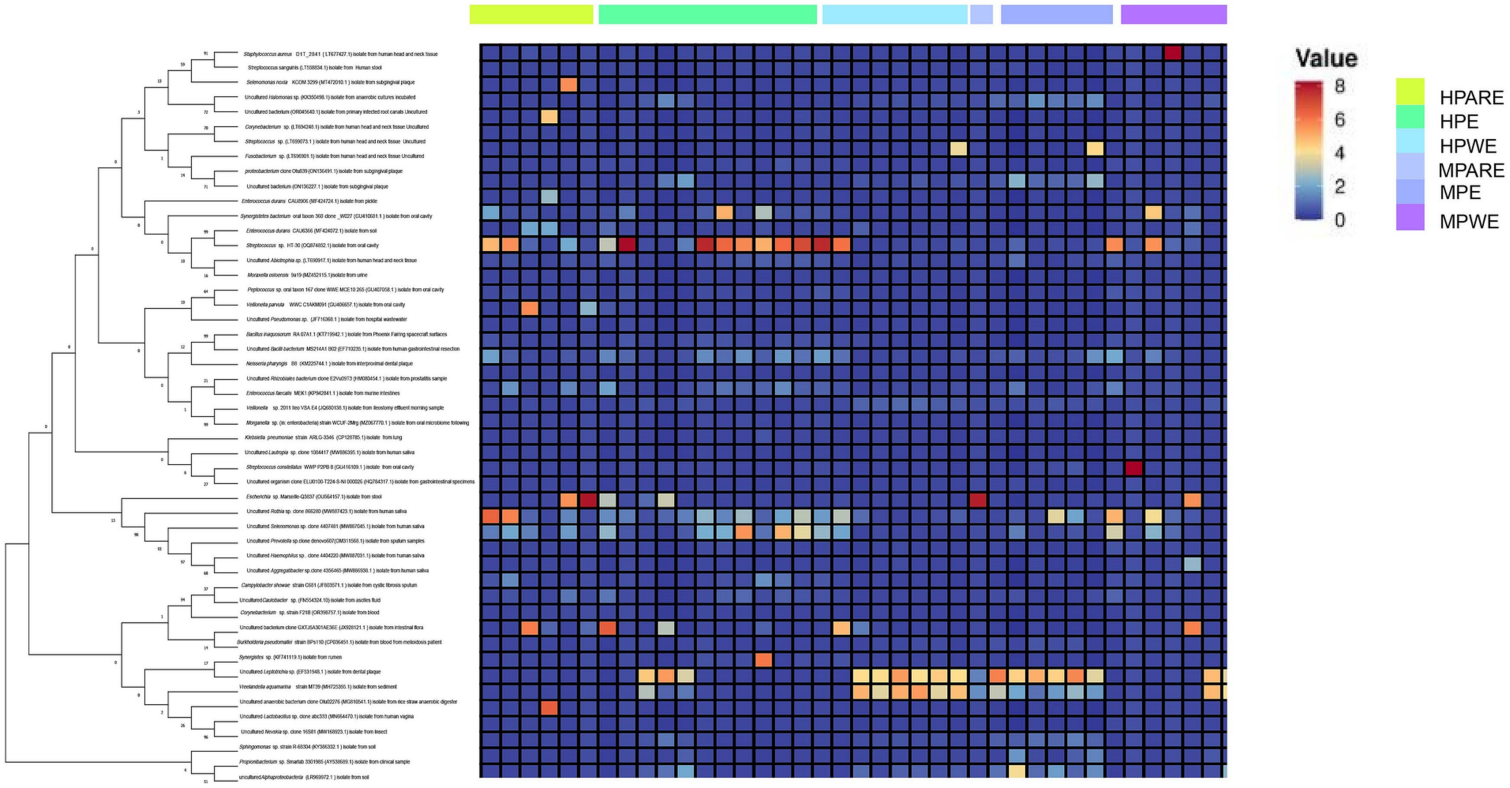
Combined diagram of heatmap and phylogenetic tree were constructed for the most abundant 50 OTUs, hydrothorax and mouthwash of different groups were, respectively, show with different colors.

The hydrothorax and mouthwash selected in this study were very useful for studying the relationship between the pleural cavity and the oral microorganisms investigated. To gain further insights into the community memberships of different types of empyema, the classified OTUs taxonomically and then compared the average abundances of each sample’s bacterial communities. The representative OTU of each type of sample was used to construct a phylogenetic tree for overall comparison. The clustering of major OTUs on the phylogenetic trees clearly showed that the hydrothorax samples were clustered together. Detailed information and specific distributions for the top 50 OTUs in all samples were shown in Figure 3. There were differences in the distribution of major OTUs among the PE group, PARE group and PWE group. The main OTU0001, OTU0002, OTU0003, OTU0004, OTU0005, OTU0006, OTU0007, OTU0008, OTU0009 and OTU0010 in the pleural cavity and oral cavity were classified as *Sphingomonas* sp., *Enterococcus durans*, uncultured bacterium isolate from intestinal flora, *Streptococcus sanguinis*, *Neisseria pharyngis*, *Burkholderia pseudomallei*, *Streptococcus constellatus*, *Klebsiella pneumonia*, *Escherichia* sp. and *Campylobacter showae*. *Streptococcus sanguinis* was detected in a large number of hydrothorax samples and mouthwash samples. *Enterococcus durans* and *Campylobacter* were mainly distributed in the PARE group and PWE group, while *Streptococcus sanguinis*, *Neisseria pharyngis* and *Escherichia* sp. were more abundant in the PE group. *Burkholderia pseudomallei*, *Klebsiella pneumonia* and *Staphylococcus aureus* were mainly distributed in the PWE group.

**Figure 4:**
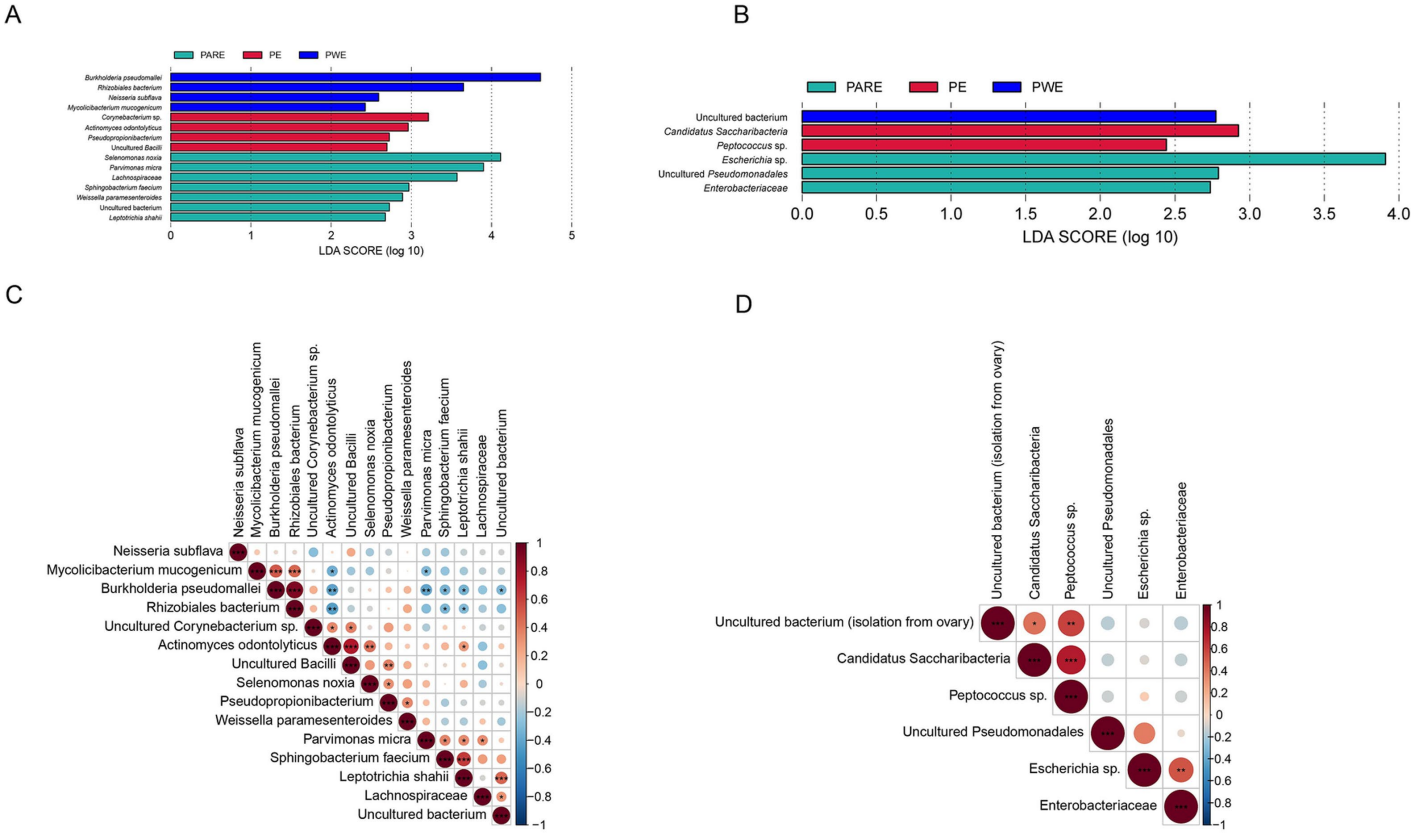
Correlation analysis of microbial markers between different group. **(A)** LEfSe analysis detected signature microorganisms at hydrothorax. **(B)** LEfSe analysis detected signature microorganisms at mouthwash. LDA > 2, red shows the positive LDA score indicating enrichment in PE samples; green shows the positive LDA score indicates the taxa enriched in the PARE sample. Blue shows the positive LDA score indicating enrichment in PWE samples. **(C)** Spearman’s rank correlation analysis of microbial markers in the hydrothorax. **(D)** Spearman’s rank correlation analysis of microbial markers in the mouthwashes. Red indicates positive correlation, blue indicates negative correlation, *p* values represented as ^*^p < 0.05, ^**^p < 0.01, ^***^p < 0.001.

Considering that there were statistical differences in *α*-diversity between primary empyema and Secondary empyema, a LEfSe analysis was performed to identify the potential bacterial candidates as biomarkers associated with empyema disease. As shown in Figure 4A. A total of 15 different species were identified in the hydrothorax, of which uncultured *Corynebacterium* sp. *Pseudopropionibacterium*, uncultured *Bacilli*, uncultured *Corynebacterium* sp., a *rhizobiales bacterium, Pseudopropionibacterium* and uncultured *Bacilli* were enriched in the PE group, *Burkholderia pseudomallei*, *Actinomyces odontolyticus*, *Neisseria subflava* and *Mycolicibacterium mucogenicum* were enriched in the PWE group, *Selenomonas noxia, Parvimonas micra, Lachnospiraceae, Sphingobacterium faecium*, *Leptotrichia shahii*, *Weissella paramesenteroides* and uncultured bacterium (isolate from *human saliva*) were enriched in the PARE group. Six different species were identified in the mouthwash samples, among which uncultured bacterium (isolation from ovary) were enriched in the PWE group, *Escherichia* sp., uncultured *Pseudomonadales* and *Enterobacteriaceae* were enriched in the PARE group, *Candidatus Saccharibacteria* and *Peptococcus* sp. were enriched in the PE group (Figure 4B).

### 3.4 Correlation analysis of biomarkers at different samples

Spearman correlation coefficient analysis of biomarkers detected at hydrothorax samples demonstrated that biomarkers within the same group were positively correlated, while those among three groups were negatively correlated. Specifically, in the hydrothorax, *Burkholderia pseudomallei*, *Mycolicibacterium mucogenicum* and a *rhizobiales bacterium* were positively correlated in the PWE group. *Actinomyces odontolyticus* were positively correlated with *Corynebacterium* sp. and uncultured *Bacilli*, and negatively correlated with *Burkholderia pseudomallei*, a *rhizobiales bacterium* and *Mycolicibacterium mucogenicum*. *Parvimonas micra* were positively correlated with *Sphingobacterium faecium*, *Lachnospiraceae*, *Leptotrichia shahii* and uncultured bacterium (isolate from *human saliva*) (Figure 4C). In the mouthwash, uncultured bacterium (isolation from ovary) were positively correlated with *Candidatus Saccharibacteria*, *Peptococcus* sp., *Escherichia* sp. and *Enterobacteriaceae* were positively correlated in the PARE group (Figure 4D).

### 3.5 Correlation analysis of the microbial and inflammatory indicators in empyema

**Figure 5:**
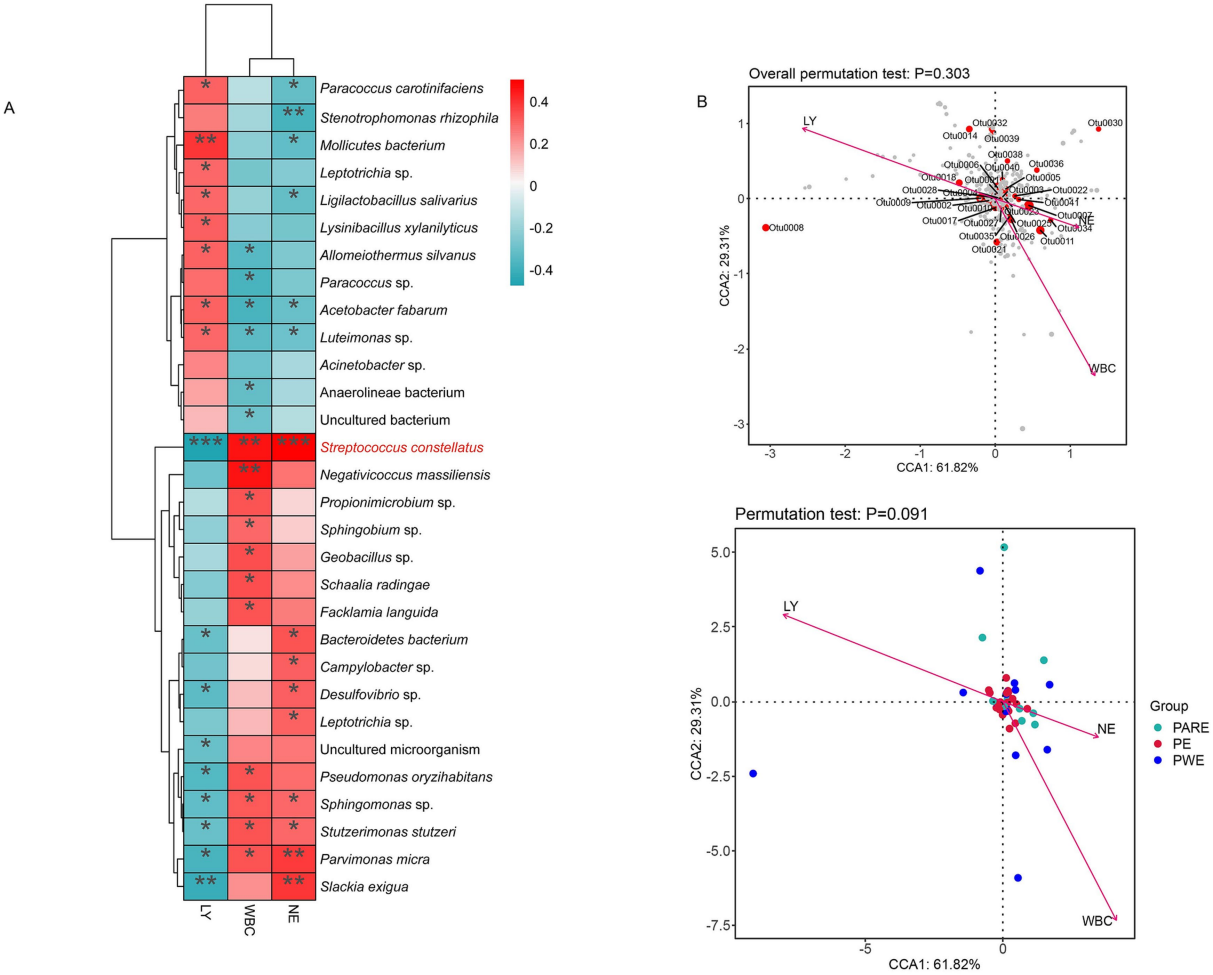
To explore the correlation between bacterial abundance and inflammatory indicators. **(A)** Correlation heatmaps represent the correlation between relative abundance of species and indicators of inflammation. **(B)** Correspondence analysis of empyema subgroup, relative abundance of bacteria and indicators of inflammation. Arrows indicate inflammation indicators; gray dots indicate different species; and the angle of the pinch indicates positive and negative correlations between inflammation indicators, acute angle: positive correlation; obtuse angle: negative correlation; right angle: no correlation.

This study conducted a correlation analysis between the inflammatory index of empyema patients and the relative abundance of bacterium using Pearson correlation analysis. The results showed that a total of 30 species were significantly associated with white blood cell (WBC) count, neutrophil (NE) ratio, lymphocyte (LY) ratio (*p* < 0.05) (Figure 5A). *Streptococcus constellatus*, *Sphingomonas* sp., *Stutzerimonas stutzeri* and *Parvimonas micra* were positively correlated with WBC and NE, and negatively correlated with LY. *Campylobacter* sp., *Bacteroidetes bacterium*, *Desulfovibrio* sp., *Leptotrichia* sp. and *Slackia exigua* were positively correlated with NE. *Pseudomonas oryzihabitans*, *Negativicoccus massiliensis*, *Propionimicrobium* sp., *Sphingobium* sp., *Geobacillus* sp., *Schaalia radingae* and *Facklamia languida* were positively correlated with WBC. *Bacteroidetes bacterium*, *Desulfovibrio* sp., uncultured microorganism, *Pseudomonas oryzihabitans*, *Sphingomonas* sp., *Stutzerimonas stutzeri*, *Parvimonas micra* and *Slackia exigua* were negatively correlated with LY. Further correspondence analyses investigated associations between indicators of inflammation and relative abundance of bacteria and subgroups. In Figure 5B the variables related to the OTUs and empyema subgroup were located close to the origin, indicating that these variables were not strongly correlated with empyema subgroup.

### 3.6 Microbial correlations between oral cavity and pleural cavity

**Figure 6:**
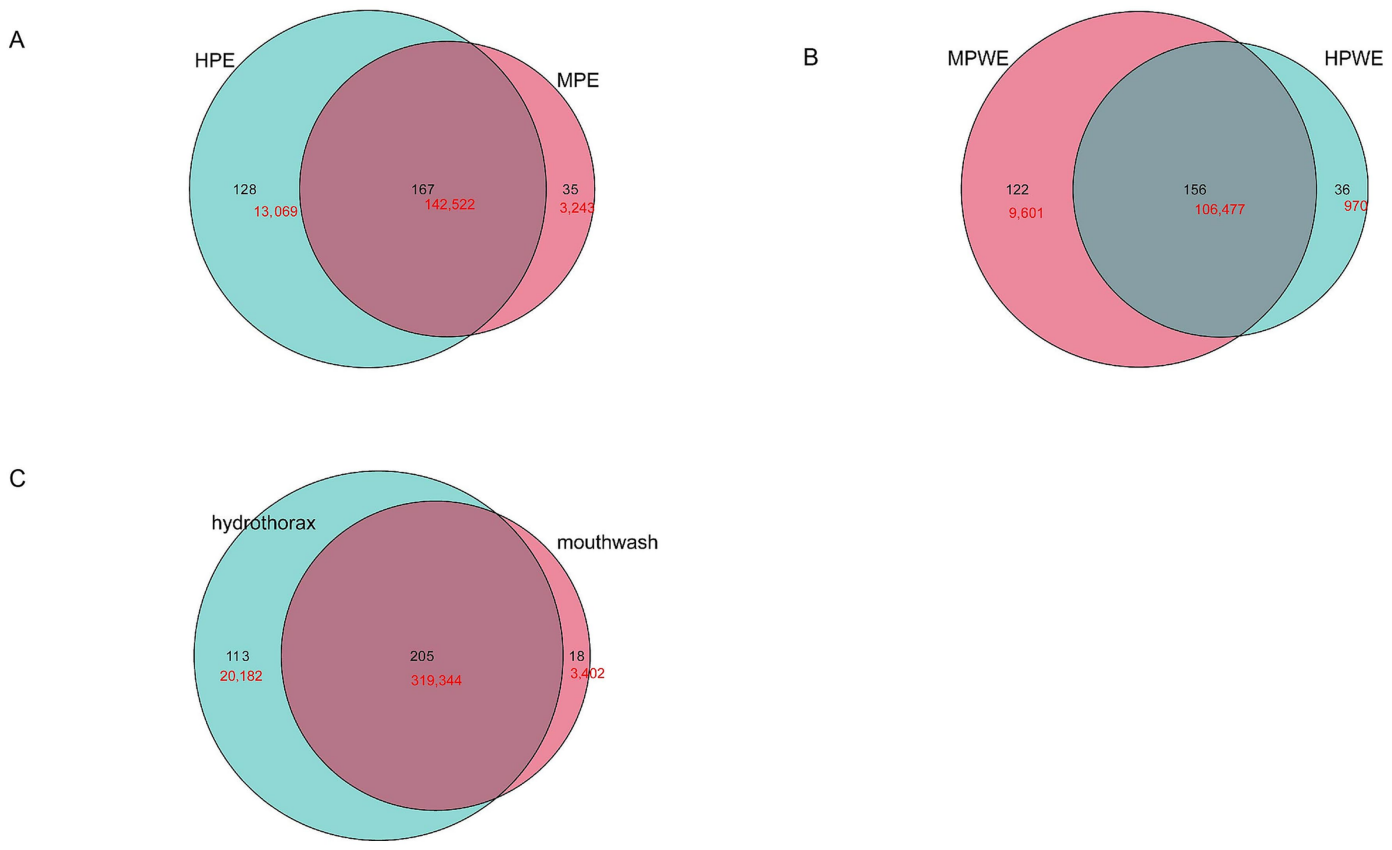
Similarity of microorganisms in the pleural cavity and the oral cavity. **(A)** Venn diagram analysis shows the OTUs and sequences shared by pleural cavity and oral cavity in PE group. **(B)** Venn diagram analysis shows the OTUs and sequences shared by pleural cavity and oral cavity in PWE group. **(C)** Venn diagram analysis shows the OTUs and sequences shared by pleural cavity and oral cavity in all empyema patients. Black shows the OTU number; red shows the sequence number.

To more precisely determine the origin of pleural cavity microorganisms, in subsequent analysis, the OTUs were determined more strictly, and the same sequences were classified as OTUs. The results revealed that the microbial patterns observed in the PE and PWE groups closely resembled those of the oral microbiota (Figures 6A,B). The shared sequences between the hydrothorax samples and mouthwash samples from the patients with empyema contributed about 90% of the total sequences used in these analyses. The shared sequences between the hydrothorax samples and mouthwash samples from the patients with empyema contributed about 94% of the total sequences used in these analyses (Figure 6C). The most frequent common species were classified as *Streptococcus sanguinis, Sphingomonas* sp.*, Neisseria pharyngis*, *Burkholderia pseudomallei, Enterococcus durans, Rothia* sp., *Campylobacter showae*, and *Escherichia* sp., all of which were detected in both sample types.

## 4 Discussion

Empyema continues to pose significant challenges in contemporary medical practice (Lee et al., 2021). The increase of complex cases was a major challenge in the treatment of empyema. Consequently, implementing patient stratification based on etiological factors could potentially elucidate these complexities, enabling more accurate prediction of causative pathogens and infection sources. This approach might facilitate the optimization of antibiotic therapy, thereby enhancing treatment outcomes.

According to the clinical observation, pleural empyema and lung abscesses might happen in the same patient. In line with the findings of the current study, Cai et al. (2019) reported that 24.8% of their study population diagnosed with empyema exhibited pulmonary abscesses. Although pulmonary abscesses might develop in individuals of any age, recent epidemiological studies have identified male gender and younger age as significant risk factors for this condition (Montméat et al., 2024). Effective host defense against bacterial invasion is characterized by the robust recruitment and activation of inflammatory cells, a process mediated by the coordinated expression of pro- and anti-inflammatory cytokines. CD4^+^ T cells, which regulate chemokine expression, play a critical role in antimicrobial host defense (Mohammed et al., 2000). Secondary empyema is frequently associated with comorbid conditions that can compromise the immune system, leading to reduced CD4^+^ T cell levels. In contrast, patients with primary empyema typically exhibit a highly activated immune system, as it has not yet been suppressed by chronic inflammation. This immunological difference might accounts for the observed lower levels of CD4^+^ lymphocytes in secondary empyema compared to primary empyema.

Empyema was a serious complication of pneumonia. Epidemiological studies indicate that approximately 60% of empyema cases are directly associated with primary pneumonia (McCauley and Dean, 2015). However, our current findings reveal a contrasting pattern, with up to 60% of empyema cases potentially originating from non-pneumonic processes. This discrepancy might be attributed to the diagnostic challenges in identifying community-acquired pneumonia (CAP) (Metlay et al., 2019), whose diagnostic criteria rely on clinical features with limited specificity and sensitivity. These clinical manifestations often overlap significantly with the characteristic signs and symptoms of pleural empyema. The researcher suggests that the documented incidence rates of pneumonia might significantly overestimate its true prevalence in the population (Hassan et al., 2019).

Pneumonia remains one of the most prevalent infections among elderly populations, a demographic characteristic particularly relevant to our study as the PWE group demonstrated the highest mean age. The clinical course of pneumonia in elderly patients is frequently complicated by coexisting cardiopulmonary comorbidities and compromised immune defenses, resulting in substantially higher mortality and morbidity rates compared to younger populations (Henig and Kaye, 2017). A consistent male predominance has been consistently documented across multiple studies investigating pleural empyema, particularly in cases associated with the *Streptococcus anginosus* group (SAG) (Grijalva et al., 2010). This gender disparity may be attributed to the higher prevalence of severe odontogenic infections observed in male patients (Cachovan et al., 2013).

In this study, Primary empyema is the most common type of empyema, which was significantly different from the sex distribution of the empyema after typical pneumonia. The same uneven gender distribution has been observed in other studies on pleural empyema, and specifically among those caused by the SAG (Cachovan et al., 2013; Grijalva et al., 2010). One explanation for the male predominance might be a higher frequency of serious odontogenic infections in male (Cachovan et al., 2013).

This study performed a comprehensive analysis of the microbial profiles across various empyema subtypes in adult populations. Primary empyema cases demonstrated significantly higher microbial species richness than secondary empyema. This observation contrasts with the characteristic microbial profile of typical pneumonia cases, which are generally associated with significantly reduced bacterial community diversity and predominant colonization by specific bacterial species (Mendez et al., 2019). Notably, antibiotic resistance patterns were prevalent across all empyema types, despite variations in their microbiological spectra. Recent evidence suggests a positive correlation between microbial diversity and the abundance of antibiotic resistance genes (Chen et al., 2021). Research has demonstrated that the differences in bacteriology are likely attributed to the acidic and hypoxic environment of the infected pleural space, which favors the growth of selected pathogens, such as obligate and facultative anaerobes (Dyrhovden et al., 2023; Maskell et al., 2006). Many of the anaerobic bacteria associated with pleural infections are strictly anaerobic and cannot tolerate the pO (2) levels of the lung parenchyma. In contrast, streptococci of the *Streptococcus milleri* group thrive in low pH and low pO (2) environments and are well-adapted to such conditions in both tissue and artificial culture settings (Whiley et al., 1992). The microbiological differences from pneumonia suggest that future studies should try to define which clinical phenotypes are associated with which pattern of pathogen.

The study by Dicker et al. (2021) clearly demonstrates that microbiomes dominated by Proteobacteria are associated with impaired lung function and increased frequency of exacerbations (Dickson et al., 2014). A significant increase in the relative abundance of the Proteobacteria phylum was also observed in the intestinal microbiota of mice with inflammation (Shin et al., 2015). This phenomenon can be attributed to the metabolic capacity of certain bacteria, particularly those within the Proteobacteria phylum, to utilize inflammatory byproducts for their survival (Scales et al., 2016). A similar mechanism may also be applicable in the context of this study.

The composition and origin of the pleural cavity flora were analyzed. The sequence of OTU0001 exhibited 98.05% similarity to *Sphingomonas* sp. Despite its low pathogenicity, it can cause severe infections. The composition and origin of the pleural cavity flora were analyzed. The sequence of OTU0001 exhibited 98.05% similarity to *Sphingomonas* sp. Despite its low pathogenicity, it can cause severe infections. Previous studies have identified consistent patterns in the site and origin of empyema caused by *Sphingomonas* sp., typically occurring in the lower lobe of the right lung or bilaterally. Moreover, the etiology of these cases has been linked to, or is presumed to result from, direct inhalation or oral bacterial translocation (Janković et al., 2023; Kumar and Norwood, 2022). The researchers identified a significant presence of oral flora-derived bacteria in the hydrothorax samples, including *Klebsiella pneumoniae*, *Rothia* sp., *Actinomyces odontolyticus*, *Veillonella* sp., and *Prevotella* sp. With the exception of *Klebsiella pneumoniae*, these bacteria are predominantly anaerobic (Iwata et al., 2023; Takayanagi et al., 2010). These anaerobic bacteria were involved in various oral infections, especially associated with periodontal infections. In addition, oral bacteria, such as *Streptococcus constellatus*, *Streptococcus* sp., *Staphylococcus aureus*, and *Fusobacterium* sp., have been identified as the primary causative agents of empyema. These bacteria are also among the most prevalent pathogens in dental apical abscesses and are frequently associated with severe periodontitis (Dyrhovden et al., 2019; Maskell et al., 2006). Katsuda et al. (2022) present direct genetic evidence that some of the bacteria in pulmonary abscesses and empyema were derived from the oral flora. This finding provides strong evidence for the importance of oral care in preventing pulmonary abscesses and empyema.

The sequence of OTU0004 exhibited 99.35% similarity to *Streptococcus sanguinis*. This species was detected in nearly all samples analyzed in this study, with a predominant presence in the PE group. *Streptococcus sanguinis* has been widely reported as one of the most common isolates across diverse geographical regions, particularly in cases of community-acquired infections (Hassan et al., 2019). The sequence of OTU0006 exhibited 100% similarity to *Burkholderia pseudomallei*, with a higher relative abundance observed in the PWE group. In a study by Chen et al., *Burkholderia pseudomallei* was identified as an enriched species in patients with empyema (Chen et al., 2021). Zhong et al. (2021) identified *Burkholderia pseudomallei* as dominant in critically ill patients, whereas *Veillonella* sp., *Neisseria*, *Streptococcus* sp., and *Prevotella* sp. were found to be the primary active microorganisms in the respiratory tracts of patients with milder symptoms. Another group of bacteria associated with poor clinical prognosis and higher risk of death *Klebsiella pneumoniae*, *Campylobacter*, and *Staphylococcus aureus* were also detected predominantly in the PWE group.

*Escherichia* sp. typically resides in the gastrointestinal tract. de Paiva et al. (2016) found that *Escherichia-Shigella* was enriched in a mouse model of Sjögren’s syndrome (SS). This enrichment was associated with a pro-inflammatory state (Morgan, 2013). Cattaneo et al. (2017) found a positive correlation between the abundance of *Escherichia-Shigella* and the levels of pro-inflammatory molecules, including IL-6 and NLRP3. *Corynebacterium* sp., which includes species such as *Corynebacterium diphtheriae*, typically colonizes the upper respiratory tract and oral cavity. The association between *Corynebacterium* and inflammation has been previously documented (Kelly et al., 2022). Ridaura et al. (2018) further demonstrated an interaction between skin immunity, inflammation, and *Corynebacterium* species colonizing the skin. The increased relative abundance of *Corynebacterium* observed in this study may suggest its potential oral origin. These findings underscore the clinical significance of oral specimens, such as saliva, in monitoring and evaluating pleural cavity conditions.

From the results of the Venn diagram, it is hypothesized that the development of community-acquired empyema may be closely related to specific oral microbiota, but there is no significant correlation with the clinical subtype of empyema. Specific oral microbiota composition might be an important risk factor for community-acquired empyema. A study on community-acquired empyema revealed that common oral pathogens, such as viridans streptococci, *Streptococcus pneumoniae* and anaerobic bacteria, are the primary causative agents of empyema, providing robust evidence to support our hypothesis (Hjertman et al., 2022). In addition, the OTUs specific to each group of empyema imply that there are differences in the structure of the microbial community in patients with different types of empyema. These differences may be related to the patient’s underlying disease, immune status and other factors, which are important risk factors for the formation of different types of empyema. The potential role of rare biosphere in the pathogenesis of empyema should not be overlooked. Although very small in number, a growing number of studies suggest that they may play key roles in specific ecological niches or pathophysiological processes, such as uncultured *Pseudomonadales, Enterobacteriaceae*, *Escherichia* sp. and *Peptococcus* sp. In the LEfSe between groups, *Peptococcus* sp. was highlighted in the primary empyema group, uncultured *Pseudomonadales, Enterobacteriaceae*, *Escherichia* sp. was highlighted in the PARE group. This suggests that rare biosphere, although in low abundances, might still be involved in inflammatory responses, host immune responses, the colonization and infection processes of pathogenic bacteria.

The sequence of OTU0007 showed 99.35% similarity to *Streptococcus constellatus*. *Streptococcus constellatus* was an opportunistic pathogen and one of the SAG. A potential mechanism for co-infection of SAG with other anaerobes has been reported to be that anaerobes and their metabolites inhibit host bactericidal activity and stimulate the growth of SAG by impairing the function of polymorphonuclear leukocytes (Noguchi et al., 2021). The increasing number of reports of life-threatening SAG infections suggests that its virulence potential has been underestimated (Pilarczyk-Zurek et al., 2022; Xia et al., 2021). This was consistent with the results of this paper that *Streptococcus constellatus* was significantly correlated with inflammatory factors.

The strengths of this study were the first systematic assessment of the differences in microbial composition of different types of empyema and the use of percutaneous fine-needle aspiration for direct collection of hydrothorax, which completely excludes the possibility of oral contamination. However, due to the retrospective study, hydrothorax was not collected from the non-infected population, and secondly, the inclusion number was small and the sample was not completely matched with the mouthwash sample, which had limited representation in the whole population. Future studies should collect more samples from more medical centers to deepen our understanding of the microbial ecology of the pleural cavity in empyema.

## Data Availability

The original datasets are publicly accessible. This data can be found in the National Omics Data Encyclopedia (NODE) and the China National Genomics Data Center (NGDC), under accession number OEP00005999.
